# Deciphering the role of NcRNAs in Pancreatic Cancer immune evasion and drug resistance: a new perspective for targeted therapy

**DOI:** 10.3389/fimmu.2024.1480572

**Published:** 2024-11-01

**Authors:** Yu Gong, Desheng Gong, Sinian Liu, Xiangjin Gong, Jingwen Xiong, Jinghan Zhang, Lai Jiang, Jie Liu, Lin Zhu, Huiyang Luo, Ke Xu, Xiaoli Yang, Bo Li

**Affiliations:** ^1^ Department of Sports Rehabilitation, Southwest Medical University, Luzhou, China; ^2^ Department of General Surgery (Hepatopancreatobiliary surgery), The Affiliated Hospital of Southwest Medical University, Luzhou, Sichuan, China; ^3^ Academician (Expert) Workstation of Sichuan Province, Metabolic Hepatobiliary and Pancreatic Diseases Key Laboratory of Luzhou City, The Affiliated Hospital of Southwest Medical University, Luzhou, Sichuan, China; ^4^ General Surgery Department, The TCM Hospital of Longquanyi, Chengdu, China; ^5^ Department of Pathology, Xichong People’s Hospital, Nanchong, China; ^6^ Department of Anesthesia, Southwest Medical University, Luzhou, China; ^7^ Clinical Medical College, Southwest Medical University, Luzhou, China; ^8^ Department of Oncology, Chongqing General Hospital, Chongqing University, Chongqing, China

**Keywords:** tumor-immune interactions, ncRNA, tumor immune microenvironment, targeted therapy, pancreatic cancer

## Abstract

Pancreatic cancer (PC) is a very aggressive digestive system tumor, known for its high mortality rate, low cure rate, low survival rate and poor prognosis. In particular, pancreatic ductal adenocarcinoma (PADC), which accounts for more than 90% of PC cases, has an overall 5-year survival rate of only 5%, which is an extremely critical situation. Early detection and effective treatment of PC is extremely difficult, which leads many patients to despair. In the current medical context, targeted therapy, as an important strategy for cancer treatment, is expected. However, the problems of immune escape and drug resistance in PC have become two major obstacles that are difficult to be overcome by targeted therapy. How to break through these two difficulties has become a key issue to be solved in the field of PC therapy. In recent years, non-coding RNAs (ncRNAs) have continued to heat up in the field of cancer research. NcRNAs play a pivotal role in gene regulation, cell differentiation, development, and disease processes, and their important roles in the genesis, development, and therapeutic response of PC have been gradually revealed. More importantly, ncRNAs have many advantages as therapeutic targets, such as high specificity and low side effects, making them a new favorite in the field of PC therapy. Therefore, the aim of this paper is to provide new ideas and methods for the targeted therapy of PC by reviewing the mechanism of action of four major ncRNAs (circRNAs, lncRNAs, miRNAs, siRNAs) in both immune escape and drug resistance of PC. It is expected that an effective way to overcome immune escape and drug resistance can be found through in-depth study of ncRNA, bringing a ray of hope to PC patients.

## 1 Introduction

PC is a highly malignant tumor of the digestive system, characterized by high mortality, low cure rates, poor prognosis, and challenges in early detection and treatment (1, 2). Over 90% of PC cases are PADCs, which have an extremely high mortality rate and a 5-year overall survival rate of 5% (3, 4). Other PCs include pancreatic acinar cell carcinoma, cystic tumors, and endocrine pancreatic tumors, which have a lower incidence but may have a relatively better prognosis (5, 6). Approximately 65% of PC tumors are concentrated in the head of the pancreas., followed by those located in the head and tail of the pancreas (7).

Current treatments for PC include surgery, radiotherapy, chemotherapy, targeted therapy, and immunotherapy (8). Surgery is the preferred treatment for PC, but due to late diagnosis, 80% of patients lose the opportunity for treatment (9, 10). Radiation and chemotherapy can alleviate symptoms and prolong the survival cycle, but have significant side effects and are rarely able to cure patients. Targeted therapy has been a major strategy in cancer treatment research, which has the effect of enhancing therapeutic efficacy and reducing side effects (11). Immunotherapy, like targeted therapy, can specifically target tumor cells, thereby improving therapeutic efficacy and reducing side effects (12). However, both immunotherapy and targeted therapy encounter two significant challenges: immune escape and drug resistance (13).

NcRNAs, i.e. non-coding RNAs, are a class of RNA molecules with no protein-coding function (14). They play an important regulatory role in cells, affecting a variety of life activities in organisms by regulating gene expression, chromatin structure, nuclear translocation, and protein function (15, 16). In recent years, ncRNAs have attracted much attention in the field of cancer research, and are believed to play an important role in the occurrence, development, and therapeutic response of cancer (17). Meanwhile, the targeted therapeutic roles of ncRNAs have also been gradually revealed in PC.

More importantly, ncRNAs have significant advantageous features in serving as therapeutic targets:

1. ncRNAs are highly specific in their expression in tumor tissues, which means that they may become biomarkers for cancer diagnosis and prognosis or novel targets for therapy (18);
2. ncRNAs affect cancer cell proliferation, differentiation, metastasis, and death through the modulation of gene expression processes (19), so that therapies targeting ncRNAs may directly intervene in cancer progression;
3. the regulatory network of ncRNAs is complex and diverse, which provides a wealth of candidate targets for cancer-targeted therapies.

In conclusion, ncRNAs have great potential and prospects in serving as PC therapeutic targets. So in summary, in this paper, we will review the functions and regulatory mechanisms of four major ncRNAs (circRNAs, lncRNAs, miRNAs, siRNAs) in both immune escape and drug resistance to help us better understand the pathogenesis of PC, and at the same time provide new ideas and approaches for PC targeted therapy and immunotherapy.

## 2 Classification, function of ncRNAs, and their biological mechanisms in diseases

NcRNAs play important roles in gene regulation, cell differentiation, development, and disease processes (20). NcRNAs can be categorized into a number of different types, including miRNAs, circRNAs, lncRNAs, and siRNAs, depending on their length, mode of formation, and function (21).

CircRNAs are abundant, stable RNA molecules with a closed-loop structure that is conserved (22), and usually consist of hundreds of nucleotides (23). They control gene transcription through interactions with RNA-binding proteins, and also regulate signaling pathways through miRNA segregation (24, 25). They have a variety of regulatory roles in the cell, including regulation of gene expression, participation in protein synthesis, etc. (26). CircRNAs can also act as “molecular sponges” for miRNAs, adsorbing and neutralizing miRNAs, thus regulating the inhibitory effects of miRNAs on target genes (27). Through the above mechanisms, CircRNAs continuously regulates the tumor microenvironment (TME) of PC, thereby affecting the occurrence and development of cancer cells.

MiRNAs are endogenous short non-coding RNAs of 19 to 25 nucleotides in size, which are part of the epigenome. MiRNAs regulate gene expression by binding to the mRNA’s 3′-untranslated region (3′-UTR) to either inhibit translation or promote degradation of target genes (28, 29). In the process of immunization against PDAC tumors, miRNAs regulate the recruitment and activation of immune cells to tumors (30, 31). At the same time, miRNAs can also act as oncogenes or oncogenes to participate in the genesis and development of PC (32).

LncRNAs are novel non-coding RNAs over 200 nucleotides in length and are key players in tumorigenesis and immune response (33, 34). They also have multiple regulatory roles in the cell, including regulation of gene expression, participation in cell differentiation, proliferation and apoptosis, etc. (35–37). LncRNAs can bind to proteins to form ribonucleoprotein complexes, thereby regulate the activity, localization and degradation of proteins, etc. (38–40). LncRNAs can also act as oncogenes or oncogenes in PCs to participate in the genesis and development of PCs (41). Like circRNAs, lncRNAs also have the function of sponging miRNAs to inhibit the abundance and activity of miRNAs (42).

SiRNAs are short interfering RNAs, usually consisting of 21-25 nucleotides (43). They are mainly used in cells to interfere with the replication and expression of exogenous viruses or transposons, and thus protect the cells from viral or transposon infection (44). SiRNAs can bind to exogenous mRNAs, induce their degradation, and thus inhibit their translation (45). Meanwhile, siRNAs can also be used to treat PC. By binding siRNAs to liposomes, siRNAs can be introduced into PC cells, which can inhibit the growth and spreading of PC cells (46).

PiRNAs are a unique class of non-coding RNAs, usually 21-35 nucleotides in length, related to the Piwi subfamily of Argonaute proteins in animals, especially in germline cells, used to suppress transposons and maintain genome integrity. Sex (47) (48). Compared to other ncRNAs such as miRNAs, lncRNAs, and circRNAs, their functions in cancer, including pancreatic cancer, are unclear. The main function of piRNA is to silence transposable elements through multiple mechanisms (49), including retrotransposons. By forming complexes with Piwi proteins, piRNAs can target transposon transcripts for degradation or translational repression, thereby limiting their proliferation and preventing their deleterious effects on the genome (50). This transposon silencing activity is critical for maintaining germline integrity and preventing genetic instability. In addition to their role in transposon silencing, piRNAs are also involved in other biological processes such as genesis, epigenetic regulation (51). Recent studies have also shown that piR-162725 may regulate the proliferation, migration and invasion phenotype of PADC, and may also regulate the EMT, cell differentiation and metabolism of PADC (52). However, the exact mechanisms and contributions of piRNAs to these processes remain largely unclear and are an active area of ​​research.

In conclusion, ncRNAs play important regulatory roles in organisms; they can regulate gene expression levels, participate in protein synthesis, and regulate cell differentiation, proliferation, and apoptosis. An in-depth understanding of the classification and biological mechanisms of ncRNAs can help to better understand their regulatory mechanisms in PC TIME.

## 3 NcRNAs are involved in the immune response of tumor cells

**Figure 1:**
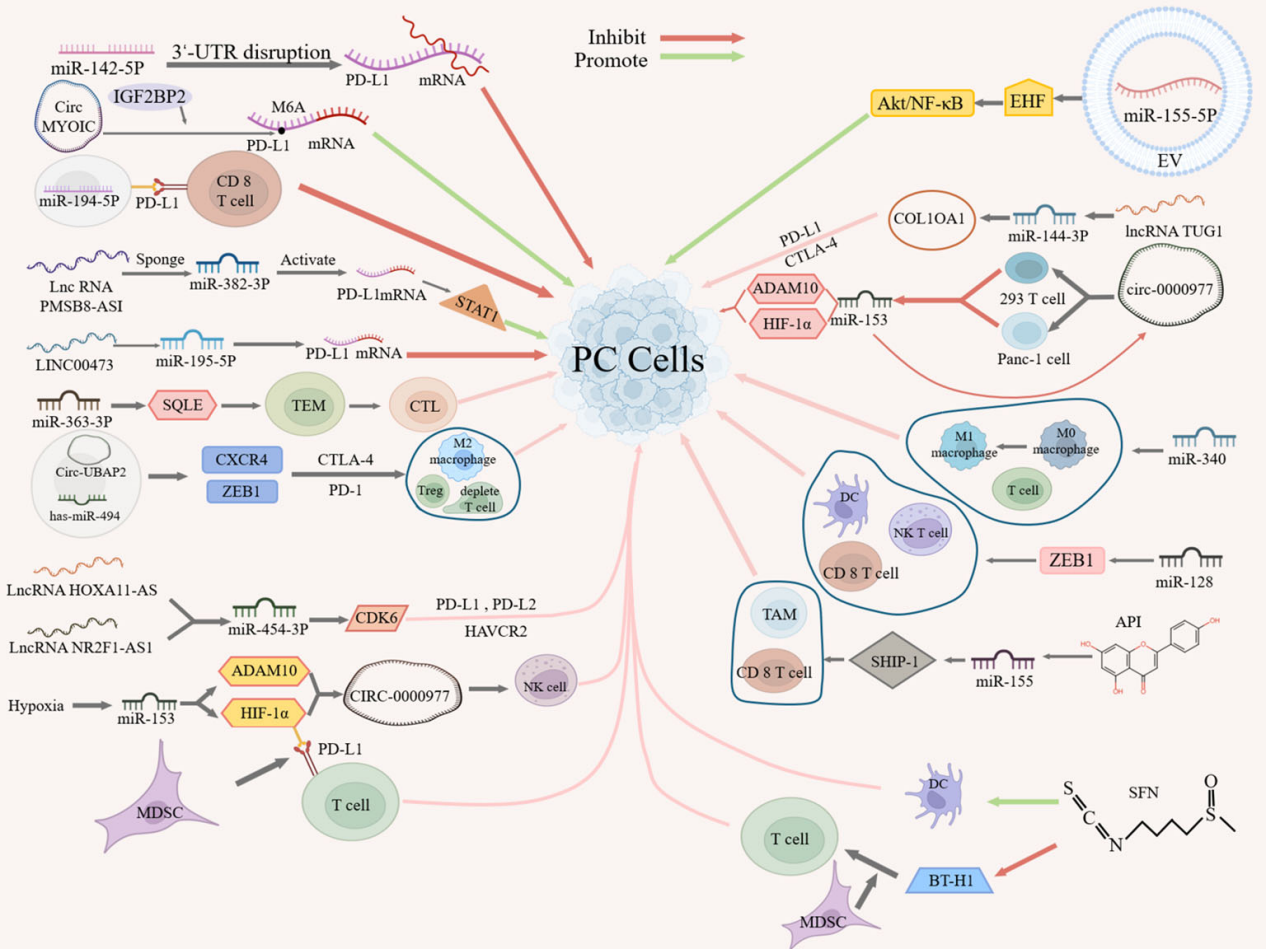
Role pathways of ncRNAs in the PC tumor immune response.

In TIME, tumor cells often evade immune cells, thus affecting the effect of immunotherapy. Understanding the mechanism of ncRNA’s action on immune escape in PC will help us better improve the effect of PC immunotherapy. Studies have shown that ncRNAs can mediate immune response through the following several mechanisms (**Figure 1**).

On the one hand, ncRNAs can affect the interaction between tumor cells and immune cells by targeting or regulating the expression or signaling of PD-L1, thereby suppressing immune response or inducing immune tolerance. For example, miR-142-5p can regulate PD-L1 expression in PC cells by binding to the 3’UTR of PD-L1 to promote tumor immune response (53). Similarly, circMYO1C can enhance the stability of PD-L1 mRNA by targeting the N6-methyladenosine(M6A) site of PD-L1 mRNA in conjunction with IGF2BP2, thereby accelerating the immune escape of PDAC (54). On the contrary, miR-194-5p enhances the toxic effect of CD8 T cells on PC cells by targeting PD-L1, thereby inhibiting the proliferation, migration and invasion of PC cells, but its specific mechanism of action remains to be investigated (55).In addition, lncRNAs can act as miRNA “molecular sponge” to target PD-L1 expression. For example, lncRNA PMSB8-AS1 acts as a miRNA sponge, which activates PD-L1 expression by interacting with and repressing miR-382-3p to regulate the transcription factor STAT1 to mediate immune escape (56). Similarly, as a miRNA sponge, LINC00473 silencing blocks PC progression by enhancing PD-L1 down-regulation against miR-195-5p (57).

Besides, some studies have shown that ncRNAs associate with immune checkpoints and indirectly influence the expression of other immune checkpoints (e.g., CTLA-4, PD-L2, HAVCR2, etc.), and may in this way mediate immune escape. For example, miR-363-3p can inhibit PANC-1 proliferation by regulating squalene epoxidase (SQLE) expression in PAAD, where SQLE regulates tumor immune cell infiltration, immune checkpoints, and the TME, and high SQLE expression predicts depletion of cytotoxic lymphocytes and loss of antitumor capacity (58). Therefore, miR-363 -3p may regulate immune checkpoints through SQLE and thus inhibit immune escape. A more definitive study has also shown that circ-UBAP2 and has-miR-494 may regulate the expression of CXCR4 and ZEB1, which are positively correlated with the expression of CTLA-4 and PD-1, and thus affect the levels of M2 macrophages, depleted T cells, and T regulatory cells (Tregs) in PAAD tissues (59), suggesting that circ-UBAP2 and has-miR-494 may regulate immune checkpoints through CXCR4 and ZEB1 factors, which in turn inhibit antigen presentation by PCs and promote immune escape. Additionally, lncRNA HOXA11-AS/lncRNA NR2F1-AS1 may regulate cyclin-dependent kinase 6 (CDK6) expression by targeting miR-454-3p, which may have oncogenic roles in PC and is strongly associated with multiple immune cells and cellular infiltration and three immune checkpoints (PD-L1, PD-L2 and HAVCR2) (60), suggesting that lncRNA HOXA11-AS/lncRNA NR2F1-AS1 may regulate immune checkpoints through the miR-454-3p- CDK6 axis, thereby mediating immune escape.

On the other hand, ncRNAs can affect the adaptation and immune escape ability of tumor cells in hypoxic microenvironments by targeting the expression or signaling of hypoxia-inducible factors (e.g., HIF-1α) or tumor suppressors. For example, hypoxia can inhibit immune escape by regulating miR-153 and its two targets, HIF-1α and ADAM10, which in turn promote the expression of CIRC-0000977, as well as by targeting PD-L1 via HIF-1α, which promotes myeloid-derived suppressor cell (MDSC)-involved T cell activation (61). CIRC_0000977 plays a role under hypoxia by regulating related pathways through miR-153, which subsequently affects the killing effect of NK cells on PC cells (61). In addition, PC-derived extracellular vesicle (EV) miR-155-5p can promote tumor immune escape by targeting the tumor suppressor EHF to downregulate, and activate Akt/NF-κB signaling (62).

In addition, ncRNAs can influence interactions in the TME by targeting components or structures of the extracellular matrix or by directly targeting cells, thereby mediating immune infiltration and immune escape. In PAAD, as a structural component of the extracellular matrix, X-type alpha 1 (COL10A1) expression was significantly up-regulated in PC tissues and significantly correlated with immune infiltration, and with immune checkpoints (PD-L1 and CTLA-4), whereas the lncRNA TUG1/miR-144-3p/COL10A1 axis was identified as the most promising upstream ncRNA regulatory pathway, suggesting that lncRNA TUG1/miR-144-3p may influence immune escape by targeting structural components of the extracellular matrix (63). In addition, cicr_0000977, a sponge of miR-153, counteracted the inhibitory effect of miR-153 on HIF-1α and ADAM10 by directly targeting 293T and Panc-1 cells, whereas miR-153 inhibition on HIF1A or cicr_0000977 knockdown on HIF1A-mediated immune escape from PC cells had the opposite effect, i.e. miR-153 inhibition partially attenuated the effect of cicr_0000977 knockdown (61). Similarly, miR-340 and miR-128 can enhance anti-tumor immunity by targeting immune cells. MiR-340 overexpression promotes macrophage(TAM) polarization to an M1-like phenotype in the peripheral and tumor-immune microenvironment, increases the number of T cells, especially CD8 T cells, contributing to the anti-tumor effects of miR-340, thereby counteracting immune escape (64). MiR-128 overexpression, in turn, modulates the percentages of dendritic cells (DCs), CD8 T lymphocytes, and natural killer T cells (NKTs) in tumors and spleens via PDAC zinc-finger E-box-binding homology cassette 1 (ZEB1) thereby enhancing anti-tumor immunity (65).

Moreover, bioflavonoids like apigenin (API) and sulforaphane (SFN) also play a significant role in regulating the immune response through their interaction with miRNAs. For example, API can promote the expression of inositol 5’-phosphatase-1 (SHIP-1) by inhibiting miRNA-155, which leads to the expansion of tumor-killing TAMs and CD8 T cells and promotes the anti-tumor immune response (66). On the other hand, SFN can enhance DC phagocytosis, and can also promote the immune response by decreasing the expression of B7-H1 and MDSC frequency in monocytes exposed to glioma-conditioned medium to reduce immunosuppression and promote T-cell proliferation (67). Among them, the reduction of B7-H1 expression not only relies on the down-regulation of STAT3 phosphorylation by SFN, but also on the up-regulation of miR-194-5p by SFN, but the miR-194-5p signaling pathway of miR-194-5p needs to be further investigated (67).

All in all, NcRNAs play a crucial role in the immune response. They can affect the immune response by influencing the activity, function and proliferation of immune cells in a variety of ways. A deeper understanding of ncRNAs will help us better understand the working mechanism of the immune system and may provide new targets for future immunotherapy and drug development. However, we still have many unanswered questions about the role of ncRNAs in immune cell regulation that require further research and exploration.

## 4 NcRNA and drug resistance

In the last 3 years, a large number of ncRNAs have been found to be involved in PC drug resistance, promoting or inhibiting the resistance of tumor cells to chemotherapeutic drugs. These findings suggest that ncRNAs may become novel targets for PC immunotherapy (**Table 1**).

**Table 1:** Mechanisms of ncRNAs in PC drug resistance.

ncRNA	Expression	Genes and pathways	Role	References
miR-3173-5p	⎯⎯	ACSL4	Promote GEM resistance and inhibit iron death	(68)
miR-378	⎯⎯	⎯⎯	Reduce GC resistance	(69)
lncRNA DSCR9	downregulated	miR-21-5p/BTG2	Affect the proliferation, invasion and GEM resistance of PC	(70)
lncRNA GAS2	downregulated	miR-21	Regulate GEM resistance in PC	(71)
lncRNA SH3BP5-AS1	upregulated	miR-139-5p/Wnt/CTBP1	Promote GEM resistance and tumor invasiveness in PC	(72)
LINC 02432	upregulated	miR-98-5p/HK2	Predict the sex of EGFR、MEK and ERK inhibitors	(73)
lncRNA UPK 1A-AS 1	upregulated	IL8, Ku70, Ku80	Confer paril-IL 8-dependent oxaliplatin resistance derived from CAF	(74)
LINC00460	⎯⎯	PDAP1/PDGFA/PDGFR/CAF	Affect GEM resistance of PADC	(75)
circACTR2	downregulated	miR-3-221p/PTEN/PI3K/AKT	Reverse GEM chemoresistance in PC	(76)
Hsa_circ_000740	downregulated	has-miR-6509-3p/DEmRNA	Mediate GEM resistance in PC	(77)
siRNA	⎯⎯	COL8A1	Inhibit the growth, migration, invasion and GEM resistance of tumor	(78)
siSLC7A11	⎯⎯	Glutathione/DOX P-glycoprotein/Bcl-2/Bax	Promote drug sensitization	(79)

MiRNAs play an important regulatory role in PC drug resistance. For instance, miR-3173-5p, which originates from cancer-associated fibroblast (CAF) exosomes in PADC, mediates gemcitabine (GEM) resistance in PC by targeting ACSL4 (68). Interestingly, miR-3173-5p also can inhibit iron death, resulting in the emergence of GEM resistance in PCs (68). Additionally, it is found that targeting inhibition of miR-378 or glucocorticoid receptor signaling can reduce PDAC glucocorticoid resistance (69).

In addition, lncRNA-miRNA interactions influence PC resistance, e.g., the lncRNA DSCR9, which is significantly down-regulated in PAAD, targets BTG2 by binding to miR-21-5p, which in turn affects PC proliferation, invasion, and GEM resistance (70). Similarly, lncRNA GAS2, significantly down-regulated in GEM-resistant PAN-5 and CaPa-1 cells, may regulate GEM resistance in PC through miR-21; however, its specific mechanism of action remains unclear (71). In addition, lncRNA SH3BP5-AS1, which is up-regulated in GEM-resistant PC cells, activates the expression of CTBP1 in Wnt pathway through competitive binding of ceRNA to miR-139-5p, which promotes GEM-resistant PC cells and tumor invasiveness (72).Moreover, Glycolysis-related LINC 02432 up-regulated in PAAD predicts the activity of PAAD patients against EGFR、MEK and ERK inhibitors by regulating hsa-miR-98-5 p/hexokinase 2 (HK2) axis (73). Besides, up-regulated lncRNA UPK 1A-AS 1 in PC promotes DNA double-strand break repair by regulating the interaction between repair proteins Ku 70 and Ku 80, thus conferring paril-IL 8-dependent oxaliplatin resistance derived from CAF (74). In addition to interacting with miRNAs, LINC00460 also regulates CAF proliferation through the PDAP1/PDGFA/PDGFR signaling pathway, thereby mediating GEM resistance in PADC cells (75).

Ibid, circRNAs also play an important role in PC GEM resistance. CircACTR2 expression is significantly down-regulated in GEM-resistant PC cells, whereas its overexpression can target the PTEN-mediated PI3K/AKT signaling pathway via sponge miR-3-221p to reverse the chemoresistance of PC cells to GEM (76). Similarly, Hsa_circ_0007401, which is down-regulated in PC-resistant cells, acts as a “sponge” of has-miR-6509-3p and thus regulates differential messenger RNA (DEmRNA) (FLI1) to mediate PC GEM resistance (77).

In addition to this, siRNAs are also involved in the regulation of PC drug resistance. SiRNA or lentiviral sh-mediated down-regulation of collagen COL8A1 in PDAC cells inhibited tumor growth, migration, invasion, and GEM resistance (78). It is also found that a ferrous organometallic framework based nanoparticles (FMN) catalyzes the iron-dependent Fenton reaction and inhibits siSLC7A11-mediated upstream glutathione synthesis to carry out intracellular self-amplified iron death, which results in a reduction of DOX-retained P-glycoprotein activity and modulation of Bcl-2/Bax expression to reverse apoptosis-resistant state of tumor cells and promote drug sensitivity (79).

## 5 Discussion

Above, we revealed the role of four major ncRNAs (circRNAs, lncRNAs, miRNAs, and siRNAs) in the PC immune microenvironment in terms of both immune response and drug resistance, indicating that ncRNAs are expected to be novel potential targets for PC targeting and immunotherapy. And currently, the function and application of ncRNAs are still advancing rapidly. A recent study also synthesized a TME stimulation-responsive poly(beta-amino ester)s (PBAE)-based polymer nano-prodrug (miR-21i@HA-Gem-SS-P12), which can co-deliver miR-21 siRNA and GEM to achieve the combination therapy of miR-21 siRNA and GEM, and shows excellent tumor inhibitory effect *in vitro* and *in vivo* in PDAC (80). Therefore, it is just around the corner for ncRNA to become the target of PC immunotherapy.

Nevertheless, there are still many challenges and problems in the practical research and application of ncRNAs. For instance, the stability, selectivity, and specificity of ncRNAs *in vivo* require further improvement. Additionally, the interactions between ncRNAs and tumor-associated immune cells need to be more clearly understood—such as how miR-194-5p targets PD-L1 to regulate immune cells (55). Further exploration is also needed on the synergistic or antagonistic effects between ncRNAs and other signaling pathways.

Therefore, this paper suggests that future research should be deepened and expanded in the following aspects:

1. Development of Advanced Detection and Analysis Methods: Establish more precise and effective ncRNA detection and analysis techniques to enhance their diagnostic and prognostic value in PC;
2. Elucidation of ncRNA-Immune Cell Interactions: Delve deeper into the mechanisms of ncRNA-tumor-associated immune cell interactions to optimize their synergistic effects in PC immunotherapy;
3. Exploration of ncRNA-Signaling Pathway Crosstalk: Investigate the intricate interplay between ncRNAs and other signaling pathways to refine strategies for comprehensive PC therapy.

By focusing on these key aspects, we can harness the full potential of ncRNAs in the fight against pancreatic cancer.
